# Invisible population: Understanding recruitment barriers of a nurse‐led support programme for families with caregiving children in Austria

**DOI:** 10.1002/nop2.491

**Published:** 2020-04-16

**Authors:** Martin Nagl‐Cupal, Julia Hauprich

**Affiliations:** ^1^ Department of Nursing Science University of Vienna Vienna Austria

**Keywords:** evaluation of services, family caregiving, family support, qualitative research, recruitment barriers, young carers

## Abstract

**Aims:**

To evaluate why no families could be recruited for a nurse‐led and family‐centred support programme in Austria which aimed to prevent an age‐inappropriate caring role for young carers.

**Design:**

A qualitative study incorporating qualitative e‐interviews and telephone interviews.

**Method:**

Twenty‐one interviews were conducted with statistically significant project stakeholders (*N* = 17) and with adult family members of children with caring responsibilities (*N* = 4). Data collection and analysis were guided by the “Social Marketing Framework.” Relevant statements were assigned to the main categories: product; price; promotion; place; and working with partners.

**Results:**

The lack of awareness towards young carers, the unfamiliar, open outcome approach of the intervention, the inappropriate language used in promotional materials and the families' fear of stigma while seeking support were identified as central obstacles for successful recruitment of families and implementation of the support programme.

## 1 INTRODUCTION

Coping with chronic illness at home often requires the efforts of the whole family (Rolland, 1999). However, there is a lack of recognition that children are also often involved in caring for an ill family member. These “young carers” are defined as children under the age of 19 who provide care for a chronically ill or disabled family member on a regular basis in the form of direct care, help with household tasks and support of healthy family members, including siblings (Becker, 2000). The available prevalence data on young carers vary between 2%–8% (Australian Bureau of Statistics, 2003; Hunt, Levine, & Naiditch, 2005; Leu et al., 2019; Metzing, Ostermann, Robens, & Galatsch, 2019; Nagl‐Cupal, Daniel, Koller, & Mayer (2014). Office for National Statistics, 2013). Caring at this young age often far exceeds the amount of assistance provided by children without caregiving responsibilities (Rose & Cohen, 2010; Warren, 2007) A recent systematic review underlined a broad variety of negative effects such as psychosocial impact in the form of anxiety, guilt and poor peer contact; impact on school life, with declining grades or lower education expectations; and substantial physical impact like sleeping disorders, headaches or back pain (Chikhradze, Knecht, & Metzing, 2017).

## 2 BACKGROUND

The needs of young carers have been the focus of a considerable number of dedicated support programmes, with services categorized according to their goals and intervention types (Purcal, Hamilton, Thomson, & Cass, 2012). Assistance and mitigation services aim to provide direct relief for affected children and adolescents or help them to cope through peer‐based activities such as clubs or summer camps, as well as support‐based activities such as person‐to‐person counselling or web‐based information services. The third approach, prevention services, focuses on the prevention of an age‐inappropriate caring role. These services aim to avoid children's entrance into caring and, where this role is already established, to help young carers achieve a level of caring that is not associated with negative effects (Purcal et al., 2012). This approach is usually family‐centred and takes into account the needs of both the children and other family members. However, the provision of such programmes is rare (Berggren & Hanson, 2015; Purcal et al., 2012).

### 2.1 Programme development

Compared with UK, Australia and Scandinavian countries, Austria pays little attention to young carers (Leu & Becker, 2017). Although smaller support initiatives have been established in recent years, there is a lack of measures focusing on prevention (Nagl‐Cupal, 2017). Based on the understanding that prevention is the most effective way to support young carers (Kavanaugh, Stamatopoulos, Cohen, & Zhang, 2015; Stamatopoulos, 2015b), we developed a family‐centred support programme for young carers and their families, which aimed to reduce the caring responsibilities of children involved.

The development was guided by Van Mejels' et al. framework for complex evidence‐based nursing interventions. To develop the programme, the following steps were worked out: 1. problem definition, 2. the accumulation of building blocks for designing the support programme, including (a) a literature review, (b) a problem and need analysis and (c) an analysis of the current practice (Van Meijel, Gamel, Van Swieten‐Duijfjes, & Grypdonck, 2004). Our basic assumption was that support for young carers and their families should contain an appropriate mix of formal and informal support where the nature, extent and overall responsibility are maintained by the family (Nagl‐Cupal & Hauprich, 2018) . We then identified the family group conference (FGC) as a promising strategy in nursing for meeting these requirements (De Jong, Schout, & Abma, 2014; Wright, 2008). The FGC is a participatory decision‐making model for families, involving a process that brings together the family, including the extended family and professionals in a family‐led decision‐making forum (Connolly, 1994, p. 87). In the context of caring families, a FGC can enable family members to take on more autonomous and alternative roles. The empowerment of families and commitment to an open outcome are the basic and health‐promoting values of FGCs Nagl‐Cupal & Hauprich, 2016).

Building on the process documentation of a young carers relief project (Schlarmann, Metzing‐Blau, & Schnepp, 2011), we tried to anticipate possible pitfalls in advance and addressed these by instituting a cooperation agreement with partners; training of intervention staff and encouraging a joint ownership approach among staff; establishing appropriate infrastructure; and engaging the public. A major homecare provider with experience in delivering a relief programme for young carers was chosen as the project partner and was involved in developing the planning and implementation strategies. In a multi‐stage process, the FGC was adapted (later “Family Conference Care”) and at least three meetings with the families, to take place in their homes, were planned. Twelve coordinators, all experienced homecare nurses, participated in a 3‐day training course to perform the intervention. The programme was designed as a pilot project and implemented in four out of nine Austrian provinces. If it had proved to be successful, it should have been implemented on a permanent basis in the other provinces.

### 2.2 Recruitment

It is well known that young carers often live in secrecy owing to stigma (Aldridge, 2018; Becker, 2007). The recruitment of families was therefore assumed to be the cornerstone for successful implementation. A recruitment protocol was developed and validated for feasibility by the coordinators, as well as the regional and national management boards of the project partner. Local social and healthcare providers, along with schools, were involved in recruitment to identify potential participants and to inform them about the programme. The recruitment phase was accompanied by an information campaign by the research team, including advertisements in local newspapers, supermarkets and major property companies. However, after 8 months not a single family had been recruited. Four families who did not live in one of the four provinces contacted the project partner. They were referred to a suitable regional home healthcare service. Owing to the expiration of the project and the impossibility of prolonging it, recruitment was forced to a halt at this stage.

Research on evidence‐based programme implementation often neglects to include an evaluation of implementation barriers (Bina, Barak, Posmontier, Glasser, & Cinamon, 2018), with recruitment being one such barrier. (Froelicher & Lorig, 2002). Issues such as a lack of time, no interest in the study, lack of literacy, lack of professional collaboration or an inappropriate strategy for promoting the study have been identified as recruitment barriers for participants in community‐based interventions (Arora et al., 2018; Bull, Boaz, & Sjostedt, 2014; Miller, Bakas, Buelow, & Habermann, 2013; Svab & Svab, 2013) as well as in family caregiver research (Funk, 2012; Morrison, Winter, & Gitlin, 2016). Furthermore, studies that report on experiences of the recruitment of young carers and their families are more than rare. Only Kennan, Fives, and Canavan (2012) reflected on the challenges to access to a sample of young carers for a study in Ireland. They emphasized that young carers' status as an invisible population, the lack of awareness of the gatekeepers involved and their lack of identification with the term “young carers” played a statistically significant role in barring their access children for the study.

As no comparable study exists in the context of young carers and their families and reporting of evaluation results for interventions that are not successful are rare, we formulated the following research question: What were the reasons why no families could be recruited for the presented family‐centred support programme for families with young carers? We believe that the lessons we learned could enable providers of such interventions to overcome recruitment barriers in future.

## 3 METHODS

### 3.1 Design

To understand the recruitment barriers, we used a qualitative research approach as it allows for deeper insight into the life experiences of those people involved, providing a view of their perceptions, meanings and interpretations of the phenomena or situation (Holloway & Galvin, 2017). This manuscript adheres to the Standards for Reporting Qualitative Research (SRQR) guidelines.

### 3.2 Data collection and analysis

Data collection and analysis were guided by the social marketing framework (Andreasen, 1995). Developed to address principal issues in social marking, this framework has emerged as a useful tool for the development and evaluation of the level of access of vulnerable and hard‐to‐reach groups (Kobayashi, Boudreault, Hill, Sinsheimer, & Palmer, 2013; Nichols et al., 2004). The framework consists of the definition of the potential target group, which is based on the definition of Becker (2000), as described above. The other components of the framework are product, price, place, promotion and working with partners (Nichols et al., 2004). These components are described in more detail in Table 1.

**TABLE 1:** Components of the social marketing framework (Nichols et al., 2004)

*Product:* The extent to which the intervention reflects the needs of the target group. For potential participants, it must be serious and acceptable enough to be interested in the intervention.
*Price:* The potential costs to participants not only financially, but also in terms of behaviour changes and time commitments, among others. Minimizing costs increases the likelihood of successful recruitment and commitment to the intervention.
*Promotion:* The techniques required to reach the target audience and to communicate information about the product.
*Place:* The places where the recruitment for or engagement in the intervention occurs, and where participants receive information about it.
*Working with partners:* The formal and informal community partners involved in recruitment, including referrals, the provision of space, screening and credibility.

As we could not draw on the experiences of family members themselves, qualitative e‐interviews were conducted with all participants who were identified as essential stakeholders for the duration of the project, namely the nurses as coordinators and the members of the project partner's regional management boards. E‐interviews focus on the content of the interviews and are used for groups of people with minimum time resources (Bampton, Cowton, & Downs, 2013). Participants' written responses to our questions, submitted via email, were used as data. Interviewees were asked about their experiences, with reference to their interactions with potential study participants. In addition, most interviewees participated in follow‐up telephone interviews to clarify and further discuss their written responses; these conversations were recorded and transcribed.

To validate the information provided by the nurses and the management board members, we conducted qualitative, semi‐structured telephone interviews with four healthy adult family members of children with caring responsibilities. Based on a purposive sample, these family members were recruited from previous studies with young carers and indicated their willingness to discuss the topic. We presented and discussed our findings and asked for their input to ascertain providers' accuracy in understanding non‐participation, from their point of view. We did not include young carers as participation in support programmes is primarily based on the decision of adult family members (Schlarmann et al., 2011). Every person we approached for participation agreed to do so. A total of 21 participants were interviewed, five members of the project partner's regional and national management boards, 12 coordinators and four healthy adults of families with young carers.

For each group of participants, interview questions were developed that reflected the different components as good as possible. The components of the social marketing framework served also as the thematic frame and main thematic categories for data analysis. The interviews were read by two researchers to familiarize themselves with the content. Relevant statements were extracted from each interview, deductively assigned to the main categories and appropriately labelled. The statements were then integrated into a table and paraphrased. The main category system was constantly expanded with sub‐categories, grounded in the data.

### 3.3 Ethical considerations

All participants received detailed written and verbal information prior to the study. They were informed that their participation was voluntary and that the reporting of interview data would be anonymized. All participants gave informed consent prior to participation. The study was approved by the ethics committee of the host university.

## 4 RESULTS

According to the components of the social marketing framework outlined above, the recruitment barriers can be described as follows.

### 4.1 Product

#### 4.1.1 Useful addition to existing measures

All interviewees regarded the FGC to be a valuable addition to existing support measures for young carers and their families. The coordinators reported great interest from colleagues, and they were convinced of its potential positive impact. It was considered to be a helpful tool for the provision of context‐specific support, as highlighted by one member of the partner organization's managerial board:

(…) in order to empower and relieve families and finally inform them about supportive measures available for their adapted situation. (Board member 3)

The development of a strategy and the inclusion of alternative private networks are key elements of the FGC, and the potential usefulness of such an intervention was confirmed by the mother of a young carer, who had developed her own type of conference with one of her ill husband's therapists:

And then we developed that plan. It was also for my husband, that he knew ‘OK, I know where I have to go today.’ – daycare for adults people for example. It gave us a daily routine. There was someone in the house the whole time. The neighbors, my mother or the children. But the children no more than three days a week. (Family member 4)

The woman included friends and neighbours in the plan to create breaks for herself and her children. This enabled her to get more time for herself and to go to work without a guilty conscience. This also released the children, who usually cared for their father with dementia on a regular basis, from their caregiving duties.

#### 4.1.2 Unfamiliar approach

The conference follows an open outcome approach, and communication between all participants is one of its central elements. Coordinators received training to facilitate an open outcome. However, while they said they would have felt comfortable in using the method, a conversation‐based, open outcome intervention is an unfamiliar approach for caring families. The coordinators assumed this to induce bias against the FGC approach from the family's point of view, which might have hindered their participation:

For the families it appears just as a clever‐talking method. They cannot imagine how talking works or what positive effects it can have. (Coordinator 1)

This “just talking” assessment was likely associated with other conversation‐based interventions, similar to the services provided by psychotherapists and psychiatrists where they may have repeated conversation on their situations without a concrete outcome. While this is not negative evaluation per se, it is considered very unusual in the context of caring. The coordinators also stated that the expectations of families receiving this type of assistance were primarily to access care or receive advice; these expectations were shared by some coordinators who were unfamiliar to the provision of care that did not include their usual interventions, such as support and counselling. In this context, the coordinators assume that families are prejudiced against such procedures and that there is a general lack of experience with “solution‐open” support in nursing interventions.

### 4.2 Price

#### 4.2.1 Time‐consuming

The FGC was framed as a pilot project, and for the families, it would have been free of charge. However, other factors were identified as “hidden costs,” such as the high time commitment required, which might have deterred potential participants. Based on their past experiences, the coordinators indicated that many adult carers often refused to participate in support measures, as caring itself is very time‐consuming:

Especially when they (families) feel a burden owing to caring, the extent and effort of the intervention were certainly deterrent. (Coordinator 1)

One family who was interested in the intervention withdrew their participation when the health status of the ill family member declined. All additional support, even when assumed to be helpful, was regarded as a minor priority for these families.

#### 4.2.2 Showing and admitting families' situation

The interviewees shared the perception that families with caregiving children lived their lives in secrecy and were therefore cautious in making use of interventions like ours. Participation was associated with admitting to and revealing the extent of their situation. They were further understood to be afraid of being convicted of integrating their children into caring of another family member:

That's why families often have a defensive attitude against all sorts of interventions in this regard. (Coordinator 11)

Even worse, participation was assumed to be displaying their potential inability to protect their children, which led to a fear of being criticized from their immediate environment as well as the health or social care professionals involved, who might not have the appropriate level of awareness regarding the young carers:

In the worst case (…) they (families) fear the potential interference from (the) youth welfare office. (Coordinator 3)

### 4.3 Promotion

#### 4.3.1 Inappropriate language

Information leaflets were created to have written, easily available promotion material. The leaflet covered information about the intervention aims, target group, procedure, involved organizations and how to contact them. The text was used in combination with basic graphic design elements to attract attention. However, the text was regarded as *“too scientific”* and therefore not accessible enough for a group of people who do not usually have much time to read—much less so if the material is difficult to understand.

The leaflets were also intended to facilitate families' self‐referrals to the programme. It became apparent, however, that the leaflets did not enable families to recognize themselves as the target group. Families with caring children often do not see themselves as such, as the following quote demonstrates:

I gave them the leaflet and reminded them to think about it. But then they (the family) have forgotten about it (the leaflet) just because they thought it did not apply to them. (Coordinator 5)

Another reason of non‐participation was assumed to be the programme's name. The term “conference” was regarded to be a deterrent as the German translation of the term *(“Familienkonferenz Pflege”)* implies an order of hierarchies and regulations which is, in fact, the opposite of what was intended:

Not conferencing or things like that. It is much simpler like sitting together and talk about families' situations. (Coordinator 10)

### 4.4 Place

#### 4.4.1 Implications of off‐site engagement

All the coordinators were very experienced homecare nurses, who had received special training in case management or other clinical specialties. However, we did not consider that the coordinators, as group leaders, spent comparatively little time to work on‐site with the families. This was later perceived as an aspect hindering extensive engagement in the recruitment process. The coordinators often had to deal with other projects, in addition to their daily routines as group leaders. This led to low priority being given to the intervention, and the coordinators tended to lose sight of it after a while.

#### 4.4.2 Not the right time for home visits

The places where potential participants could have been recruited were broadly diversified. However, the most common place of immediate recruitment was the families' home during visits of the healthcare professionals. Owing to organizational issues, these visits usually take place in the morning or early afternoon. Potential young carers were therefore overlooked because they were not at home at those times, as explained by one of the coordinators:

This is the time when children usually attend school and are not at home. (Coordinator 2)

Moreover, the coordinators stated that in private homes it was the patient and not the family who was considered to be the primary client. Even coordinator trainings did not have a significant impact on this view. This further contributed to the fact that young carers were overlooked.

### 4.5 Working with partners

#### 4.5.1 General lack of awareness

To access affected families, much effort was devoted to working with network partners and groups of people who were potentially in contact with young carers in their work environment, in addition to schools, family practitioners and youth, health and social care organizations. According to the recruitment protocol, coordinators were to be informed by a network partner when a family or a young carer had been identified as potential participants. Many partners were willing to work together with the coordinators, or at least be vigilant. However, as the interviews revealed not a single partner informed the coordinators about a young carer or families. One coordinator (4) cited the feedback of a social worker who had not: *“… had any contact with a young carer in his whole career.”*

This allows the conclusion that the general lack of awareness about young carers was a major recruitment barrier, despite the reasonable assumption that the network partners like general practitioners, social workers, healthcare professionals in hospitals and teachers have contact with young carers in their work environment. The implication is that these children are not recognized as such:

Understanding the phenomenon of young carers is the central precondition for facilitators in order to recognize them. (Coordinator 7)

This lack of awareness raises the need for a more general discussion of the phenomenon, both within communities and among network partners. This was further underlined by the coordinators' difficulties in addressing the topic with network partners. A more in‐depth discussion of the issue might have facilitated recruitment, as it would: *“(…) give the phenomenon a human face.” (Board member 2).*

## 5 DISCUSSION

This study sought answers to the question of why no participants could be recruited for a family‐centred support programme for families with young carers. We have learned, even with meticulous planning, significant recruitment problems can occur. A key finding highlights the *price* of participation. Minimizing the price should increase the likelihood of successful participant recruitment (Nichols et al., 2004). Chronic illness in the home can be described as requiring a process of “unending work and care” (Corbin & Strauss, 1988). Families therefore play the most important role in managing the everyday life and promoting self‐management of the ill family member (Whitehead, Jacob, Towell, Abu‐qamar, & Cole‐Heath, 2018). Considering these temporal demands, it is understandable that additional time‐consuming activities, such as our intervention, not have a high priority, especially when the benefit to the family is unclear. Family members of chronically ill patients are in a permanent state of action to deal with the unplanned changes occurring as the illness progresses and might feel “too busy”—a potential barrier to participation that has also been identified in other studies (Bull et al., 2014).

Another “price” to be paid was families' fear of confessing to the integration of their children into care. When a topic is associated with embarrassment or taboo, it becomes much harder to recruit potential participants, who may not admit to having a problem (Nichols et al., 2004). An essential element of the intervention was the inclusion of the wider family and their social network. In theory, this approach can promote cohesion and stimulate families' use of professional healthcare services (Nagl‐Cupal & Hauprich, 2016) . These potential benefits, however, does not take social pressure into account, which comes into play when families reveal their struggles and deficits and where social networks have the power to decide what is good for the family (Schout, van Dijk, Meijer, Landeweer, & de Jong, 2017). Young carers' families want to live their lives as normally as possible and often live hidden from the view of others, making access extremely difficult (Chikhradze et al., 2017; Kennan et al., 2012; McDougall, O'Connor, & Howell, 2018).

Although it did not play as significant a role as in other studies, another recruitment barrier was the additional work required of professionals during the recruitment process. Researchers often forget about the implications of participation for project partners, including the possibility that participation in the project is usually an additional task, carrying much less priority than it does for the researchers (Arora et al., 2018; Butterfield, Yates, Rogers, & Healow, 2003; Sullivan‐Bolyai et al., 2007). Daily routine, work burden and professionals' own priorities all contribute to them losing sight of the recruitment process. Viable incentives, which were not taken into account in this study, can help to overcome those barriers (Lloyd, McHugh, Minton, Eke, & Wyatt, 2017; Sullivan‐Bolyai et al., 2007).

Like in many other countries, young carers and their families in Austria are a hidden group of whom there is a low level of professional and societal awareness (Leu, Frech, & Jung, 2018). This point was made particularly salient in the collaboration with partners. Raising awareness on all system levels is the most important precondition to “see,” identify and refer young carers and their families to an existing support programme (Leu et al., 2018; Stamatopoulos, 2015a). With the exceptions of Great Britain, Australia and, to some degree, Scandinavian countries, no country has policies or procedures in place to guide support for children and adolescents with caring responsibilities. In the UK, the Care Act of 2014 requires the mandatory assessment of young carers' needs by local authorities. This, hand in hand with the establishment of a national research body on young carers, is assumed to be the most influential way of raising and maintaining awareness on a professional level (Becker, 2007; Leu & Becker, 2017). Even if the recruitment had been successful, it is unlikely that it would have been successful in a routine programme, as such an intervention needs to be socially and culturally acceptable to its participants (Nichols et al., 2004).

There is broad consensus that a whole family approach is a cornerstone for supporting young carers (Frank & Slatcher, 2009; Stamatopoulos, 2015b). However, locally as well as internationally, the implementation of such an approach is an assumption rather than a rule (Berggren & Hanson, 2015). The most important aspect to consider for young carers' families to accept support from professionals is the retention of control over the nature and the extent of support, as well as the overall caring responsibility (Nagl‐Cupal & Hauprich, 2018). This, however, seems to contradict the rationale of the general homecare system represented by agencies and service providers and necessitates a general cultural change on the provision of formal care for families in the community.

The social marketing framework was a useful tool for addressing the research question and structuring the findings. However, a precise distinction of its elements was difficult. “Awareness,” for example, is the most important precondition for working with young carers and impacts on promoting the measure as well as working with partners. The framework as presented by Van Meijel et al.'s (2004) was also a valuable guide for the development of the intervention at hand. However, the inclusion of target groups as a developmental step should not only occur in the analytic stages, but also in the design phase. Including families in this stage might have enabled the researchers to avoid inappropriate language use and to anticipate access and recruitment barriers prior to implementation (Nichols, 2002).

### 5.1 Limitations

The focus of this evaluation was on the developed intervention and largely neglected wider contexts that could have had also an impact on participation in the study. For example, we did not scrutinize if it was the right time to implement the programme, even though our project partner was very involved in refugee work at the time. For this study, we have interviewed those groups of people who had operational responsibility or were immediately involved in the support programme. This provided us with a proxy perspective, and the reasons for non‐participation of those people who decided not to participate or withdrew their decision to do so remain largely unclear. The inclusion of this perspective, however, would have presented ethical concerns as it might have increased pressure on potential participants to justify their decisions.

## 6 CONCLUSION

This study revealed the major recruitment barriers of families with caring children for a family‐centred support programme. Although the study had a clear focus on young carers' families, it could be argued that the challenges experienced in this instance are applicable to other hard‐to‐reach groups as well. It is clear that much more effort should be made in raising awareness of the existence and needs of young carers. Even if the recruitment had been successful, the permanent roll‐out of the project requires cultural change where young carers' families can participate in support programmes without the fear of being stigmatized and where nurses and other health and social care professionals acknowledge a whole family approach as a cornerstone in the support of the target group.

## 7 RELEVANCE TO CLINICAL PRACTICE

The findings of the study can enable providers of such interventions to overcome recruitment barriers in future more easily. Healthcare providers should be aware of the role of young carers within caring families. Raising awareness on all system levels is the most important precondition to “see,” identify and refer young carers and their families to an existing support programme.

## CONFLICT OF INTEREST

The author(s) declared no potential conflicts of interest with respect to the research, authorship and/or publication of this article.

## Data Availability

The data that support the findings of this study are available on request from the corresponding author, [MNC]. The data are not publicly available due to privacy and ethical restrictions.
